# Eyes Wide Shut: Amygdala Mediates Eyes-Closed Effect on Emotional Experience with Music

**DOI:** 10.1371/journal.pone.0006230

**Published:** 2009-07-15

**Authors:** Yulia Lerner, David Papo, Andrey Zhdanov, Libi Belozersky, Talma Hendler

**Affiliations:** 1 New York University, Center for Neural Science, New York, New York, United States of America; 2 Functional Brain Imaging Unit, Tel Aviv Sourasky Medical Center, Tel Aviv, Israel; 3 Tel Aviv University, Tel Aviv, Israel; University of Leuven, Belgium

## Abstract

The perceived emotional value of stimuli and, as a consequence the subjective emotional experience with them, can be affected by context-dependent styles of processing. Therefore, the investigation of the neural correlates of emotional experience requires accounting for such a variable, a matter of an experimental challenge. Closing the eyes affects the style of attending to auditory stimuli by modifying the perceptual relationship with the environment without changing the stimulus itself. In the current study, we used fMRI to characterize the neural mediators of such modification on the experience of emotionality in music. We assumed that closed eyes position will reveal interplay between different levels of neural processing of emotions. More specifically, we focused on the amygdala as a central node of the limbic system and on its co-activation with the Locus Ceruleus (LC) and Ventral Prefrontal Cortex (VPFC); regions involved in processing of, respectively, ‘low’, visceral-, and ‘high’, cognitive-related, values of emotional stimuli. Fifteen healthy subjects listened to negative and neutral music excerpts with eyes closed or open. As expected, behavioral results showed that closing the eyes while listening to emotional music resulted in enhanced rating of emotionality, specifically of negative music. In correspondence, fMRI results showed greater activation in the amygdala when subjects listened to the emotional music with eyes closed relative to eyes open. More so, by using voxel-based correlation and a dynamic causal model analyses we demonstrated that increased amygdala activation to negative music with eyes closed led to increased activations in the LC and VPFC. This finding supports a system-based model of perceived emotionality in which the amygdala has a central role in mediating the effect of context-based processing style by recruiting neural operations involved in both visceral (i.e. ‘low’) and cognitive (i.e. ‘high’) related processes of emotions.

## Introduction

The perceived emotional intensity of stimuli can determine its assigned adaptive value by the individual. It has been acknowledged that perceived emotionality of stimuli is highly dependent on style of processing (e.g. focus of attention), perceptual context (e.g. the relatedness to environment) and task relevance, which together form the individual's mental set [1]. Exploring the neural marking of a change in style of processing may reveal the underlying mechanism of the phenomenal aspect of emotional experience. However, this calls for experimental probes of shifts in style of processing while keeping unchanged the physical properties of the stimulus used as a carrier for emotions. Closing one's eyes while listening to sound may serve such aim as it evokes shifts in style of processing by modifying focus of attention, while keeping targeted stimuli the same. The main outcome of such a shift could enhance the perceived intensity of emotional stimulus, making positive attributes more positive and negative ones more negative. For instance, the positive feeling of relaxation is facilitated by closing the eyes; on the other hand, when closing the eyes in the presence of negative-valued external stimuli, subjects tend to increase their preparedness to a possible threat. Brain wise, the closed eyes position is well known by its unique electrophysiological signature of increased alpha rhythm [2]. In a recent resting state study, fMRI and EEG signals were recorded simultaneously while participants intermittently were instructed to close or open their eyes. Using the combined measurements, two alpha rhythms were defined tempo-spatially with relation to the closed eyes position; one on-going and spontaneous distributed in the midline brain regions including the prefrontal cortex (PFC), cingulate cortex and thalamus, and the other stimulated by shift in eyes position from open to closed distributed in the fronto-temporal cortical regions. These data point to the possibility that closing the eyes indeed characterizes a specific brain state that can be affected by the individual's mental set [3]. Accordingly, the current study presumes that eyes closed position represents a well defined mental set by which perceived emotionality can be modulated, thus probing its neural respect.

Changes in perceived emotionality associated with a given stimulus can be related to modification in the processing of visceral- or cognitive emotional cues, through activation of vigilance- or appraisal-based mechanisms, respectively. The importance of visceral-related cues in the primacy of emotional processing was originally suggested by James [4] and more recently updated as the ‘somatic marker hypothesis’ by Damasio [5]. On the other hand, a critical involvement of cognitive schema of appraisal in the establishment of emotional experience has been originally put forth by Schachter and Singer [6] and more recently emphasized from a clinical perspective by Beck and Clark [7]. It seems though, that people vary tremendously in their tendencies to rely on either of these processes when encountering an emotional stimulus in the environment. While some individuals described as ‘aware of their visceral cues’, tend to be more emotionally expressive and to experience emotions as more intense [8], [9], others tend to be over-occupied with internally generated self-focused negative ruminations and suffer from depressive symptoms [10]. This inter-individual variability calls for a certain interplay between visceral and cognitive based neural operations of emotion, as nicely suggested recently by the ‘conceptual act model’ [11]. This model posits that our aware emotional experience emerges out of an ongoing flux of a ‘core affective state’ (i.e. the experience of a pleasant/unpleasant feeling with a varying amount of arousal) as conceptualization (i.e. appraisal, reflection) of this state takes place. Furthermore, those two processing stages assumed to be mediated by different brain networks, involving ‘lower’ (i.e. independent of directed attention and awareness) and ‘higher’ (i.e. depends on directed attention and awareness) levels of brain operations, respectively. Interactions within and between these levels may play a key role in the establishment of the unique subjective emotional experience out of an ongoing background feeling state.

The neural apparatus mediating the processing of affective attributes of stimuli (i.e. perceived emotionality) has been extensively studied in humans, although the modulating effect of the perceived intensity has hardly been examined. The amygdala together with its dense connections with both prefrontal cortex and the brainstem has been put forth as a core region in this respect [12]. Projections of the amygdala to brainstem areas such as the LC were proposed as instrumental in the detection of and orienting to visceral signals of emotions [13]. Recent imaging studies further support the relatively automatic nature of emotional processing via this path, by showing a closer relation between the amygdala and the LC activation during unaware than aware processing of negative facial expressions [14].

The amygdala's dense connections with the VPFC have been repeatedly demonstrated in animals [15]–[17]. More recently, imaging studies demonstrated a correlation between activations in the amygdala and the PFC including its ventral aspect in relation to appraisal of negative emotional stimuli [18], [19]. Importantly, neuroanatomical and physiological studies also show that the PFC is heavily projecting to the LC. For example, the monkey LC has been shown to receive strong direct projections from various medial prefrontal regions thought to evaluate and monitor task-related contexts [20]. Accordingly, these authors proposed recently that LC might be responsive to ongoing evaluations of task-related appraisal provided by inputs from the PFC. Subsequently, LC modulates cortical sites as a function of the appraisal processes, thus selectively adjusts subjects' mental set towards task- or stimulus-specific processes. Yet, the mode of interplay between ‘low’ and ‘high’ levels of neural operations for determining the perceived emotional value is poorly understood.

**Figure 1:**
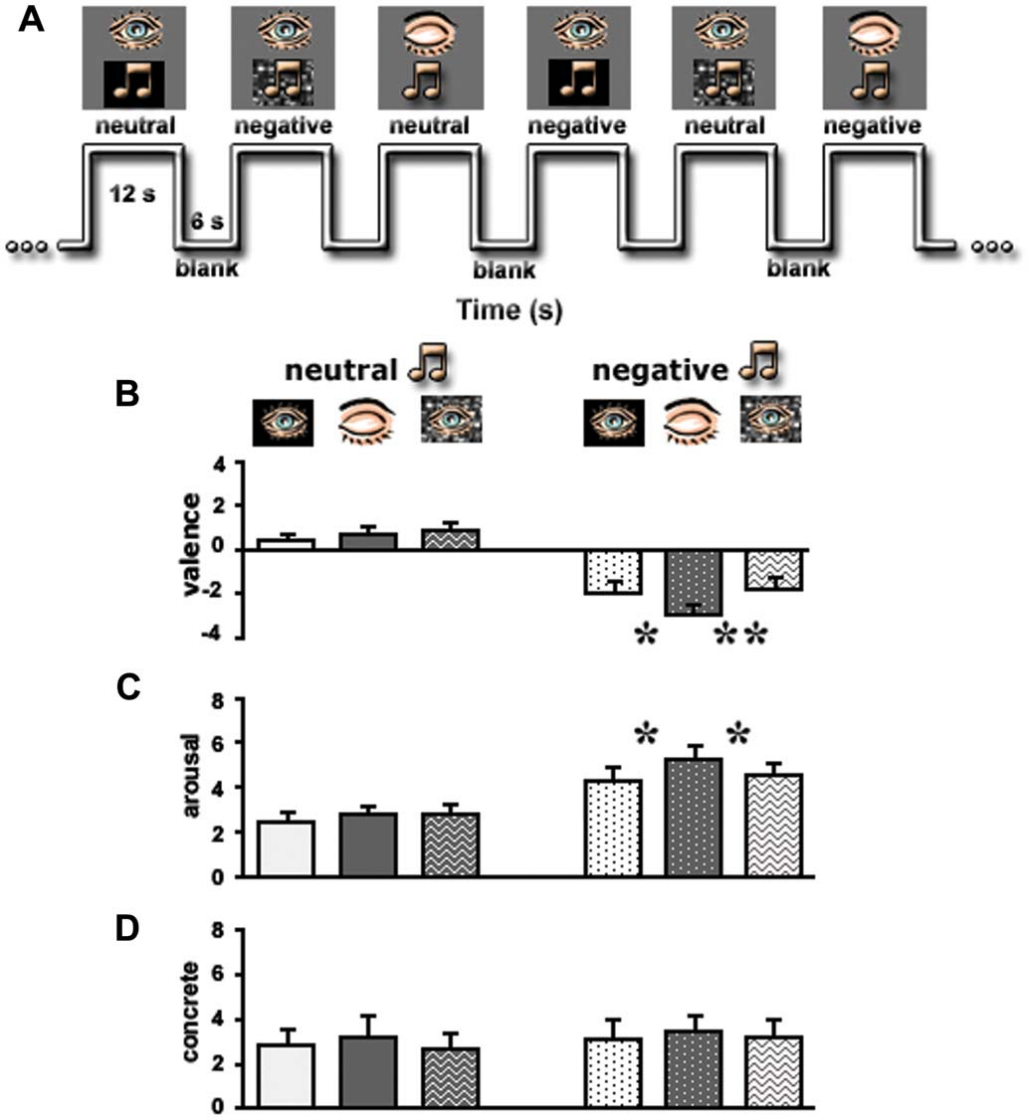
Experimental design and behavioral results. (A) A segment from the time axis of the experiment. (B)–(D) Results of psychophysical tests conducted outside the magnet on the same subjects who participated in the fMRI study and using the same stimuli. Evaluation of the emotional valence (B), arousal (C) and abstraction (D) levels of the clips are presented. Stimuli types (x-axis) are identified in the apertures above the graphs. Note a significant difference only for negative clips presented with eyes closed. Y-axes represent emotional dimensions. *, *p<0.05*; **, *p<0.01*. Error bars, SEM.

In the current study we investigated whether closing the eyes while keeping stimuli and task unchanged, modifies the perceived intensity of the emotional value conveyed by various musical excerpts. Music has commonly been used as an emotional stimulus due to its highly affective, though abstract, value in humans [21]. Interestingly, most imaging studies failed to demonstrate amygdala activation to emotion in music [22], [23], but see [24], [25]. In a recent study in our lab we found that adding emotional music to a short neutral video clip resulted in increased emotional value of the movie and greater activation in the amygdala than with the presentation of the neutral movie or emotional music alone [26]. It is not clear though if this combined presentation effect in the amygdala was due to increased semantic cues or greater complexity of the emotional stimuli (i.e. multi-modality). Here, we are able to manipulate the perceived emotionality via change in the mental set (i.e. eyes position) while keeping physical aspects of the emotional stimuli unchanged (i.e. music). This way we could test separately the effects of perceived emotional value (i.e. negative vs. neutral), processing context (eyes open vs. eyes closed), and their interaction (change in perceived intensity with regard to eyes position). For that, we performed behavioral and fMRI experiments using short music clips of negative and neutral emotional values (Stimuli S1, S2, S3, S4, S5, S6, S7, S8) presented in three context conditions (see Figure 1A): when subjects' eyes were (i) closed, (ii) open while viewing a black screen, (iii) open while viewing a scrambled video clip. In a separate fMRI experiment we examined the effect of closing and opening the eyes in complete darkness (see Materials and Methods). We assumed that the eyes closed position would enhance perceived emotional intensity of the musical excerpts, more so for the negative pieces, and that this would be associated with increased activation in the amygdala, as well as with greater functional connectivity between the amygdala, the LC and VPFC; representatives of the ‘low-visceral’ and ‘high-cognitive’ neural operations of the human emotional experience.

## Results

### Behavior

Behaviorally the experiment was set to answer to what extent closing the eyes affects the experience of emotion in music. Figure 1B–D show that there was a significant difference in valence rating between closed and open eyes states only for the negative musical clips, with a higher rating for the closed eyes state (one-tail *t*-test: ‘negative closed’ vs. ‘negative open’: t(11) = −2.1, *p<0.05*; ‘negative closed’ vs. ‘negative scrambled-movie’: t(11) = −3.2, *p<0.01*). Moreover, there was a similar difference for closed vs. open eyes for the rating of arousing value of negative music (one-tail *t*-test: ‘negative closed’ vs. ‘negative open’ and ‘negative closed’ vs. ‘negative scrambled-movie’: t(11) = −2.04, *p<0.05*). In contrast, no such difference was obtained for the rating of the level of abstraction in the stimuli.

### fMRI: Activation Distribution and Magnitude

**Figure 2:**
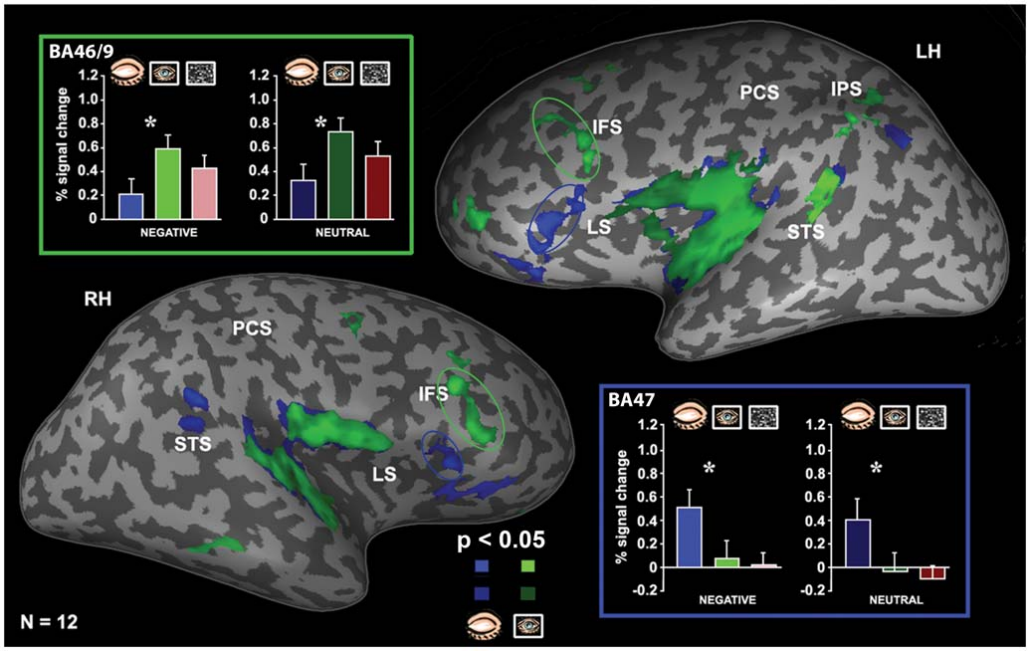
Differences in brain activation with eyes closed, eyes open and activation profiles of ROI. Functional averaged activation maps (N = 12, *p<0.05*, random effect) show the cortical activity evoked by musical clips (negative and neutral) presented with eyes closed (blue) and open (green). The maps are superimposed on the left (LH) and right (RH) unfolded hemispheres shown in the lateral view. Quantitative analysis of the activation levels is shown for open (green patches on the maps) and closed (blue patches on the maps) eyes. While the BA 47 (‘blue-framed’ bars, blue circle on the map) exhibited highly preferential activation for eyes closed, the BA 46/9 (‘green-framed’ bars, green circle on the map) demonstrated highly preferential activation for eyes open. STS – superior temporal sulcus, IPS – intraparietal sulcus, PCS – post-central sulcus, LS – lateral sulcus, IFS – inferior frontal sulcus. Error bars, SEM. *, *p<0.05*.

Analyzing the imaging data we first performed a whole brain analysis to delineate the eyes-state related networks. The results of this analysis are summarized in Table 1. Closed eyes vs. open eyes across music conditions revealed activation in the VPFC, superior temporal cortex as well as in the limbic areas such as the amygdala, anterior hippocampus, temporal pole and cingulate cortex. In contrast, open eyes vs. closed eyes showed as expected preferential activation in perceptual regions in the occipital, parietal and lateral temporal regions as well as in the dorso-lateral PFC (DLPFC). This analysis highlighted an interesting dissociation in PFC activation in regard to mental set induced by eyes position. Figure 2 shows the striking functional segregation in the PFC between eyes positions on inflated group activation map (N = 12, *p<0.05*, random effect); closed eyes were associated with activation of VPFC areas (BA 47 and BA 11), while open eyes evoked activity in the DLPFC areas (BA 46/9 and BA 10). Talairach coordinates and voxel extension are presented in Table S2.

**Table 1:** Whole-brain activation analysis.

Cortical Region	Approximate Brodmann Areas (BA)	Talairach coordinates (mm)						Peak p-value
		LH			RH			
		x	y	z	x	y	z	
	**open vs. close (random effect)**							
Early Visual Areas	17, 18	−11	−92	−3	9	−83	0	p<0.000007
Precuneus	7, 19	−14	−72	37	23	−73	30	p<0.00005
Cuneus	18, 19	−5	−80	26	6	−73	15	p<0.00005
Intraparietal Sulcus	39, 7	−31	−52	31	18	−78	31	p<0.0006
Posterior Cingulate	30	−9	−55	6				p<0.002
Superior Parietal Lobule	7				26	−56	46	p<0.0005
Superior Temporal Sulcus	22	−44	−46	19				p<0.001
Middle Frontal Sulcus	9, 46	−39	8	32	42	37	25	p<0.008
Middle Frontal Gyrus	9,10	−35	46	9	40	46	14	p<0.001
Superior Frontal Gyrus	10	−9	62	31	26	61	19	p<0.0005
Thalamus		−20	−29	0	21	−26	1	p<0.0001
	**close vs. open (random effect)**							
Intraparietal Sulcus	7	−39	−64	31				p<0.001
Superior Temporal Sulcus	22	−43	−43	21	48	−46	16	p<0.008
Orbital Sulcus	11	−19	44	−6	17	48	−5	p<0.008
Lateral Sulcus/IFG	47	−38	28	−1	32	32	−7	p<0.001
Amygdala		−24	−5	−12	20	−4	−14	p<0.0001
Hippocampus		−24	−11	−13	21	−12	−14	p<0.0001
Cingulate Gyrus	24				7	−7	44	p<0.05
Anterior Superior Temporal Gyrus	38	−45	3	−20	43	4	−14	p<0.005

Areas that exhibited the significant levels of activation during stimulation by the eyes closed and the eyes open states.

**Figure 3:**
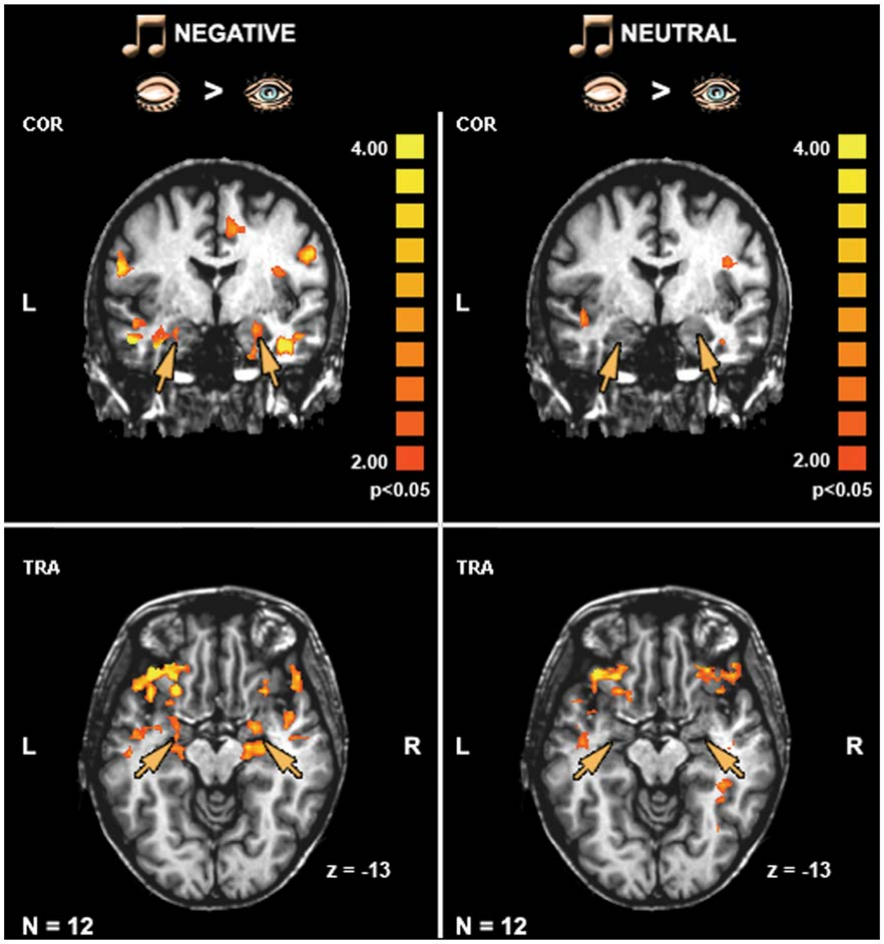
The effect of eyes closed on the amygdala. Average activation patterns (N = 12, *p<0.05*, random effect GLM analysis) revealed by the contrast ‘eyes closed > eyes open’ and superimposed on the coronal and transversal views. Significant activation was found in the amygdala/anterior hippocampus complex (orange arrows) for the negative clips (left panel) but not for the neutral ones (right panel). The color scale indicates significance level. L – left hemisphere, R – right hemisphere, COR – coronal, TRA – transversal.

Since this study was focused on the effect of shifting eyes position on emotional experience we examined the difference between closed and open eyes in relation to each music type separately (i.e. neutral and negative). Figure 3 shows the results from whole brain analysis on two views of group maps (N = 12, *p<0.05*, random effect). It can be seen that during negative music but not during neutral music closed eyes evoked greater activation than open eyes in the amygdala/anterior hippocampus complex and anterior temporal poles. This pattern was highly consistent across all subjects as shown by individual's activation maps (Figure S1). In contrast to limbic areas, increased activation in the VPFC for closed eyes relative to open eyes was seen for both negative and neutral music types.

It is interesting to note that the VPFC foci showed a slight trend toward greater selectivity to emotion when subjects were instructed to judge the emotionality of musical clips (% signal change, negative, closed: 0.62±0.26 with task vs. 0.52±0.19 without task; open: 0.16±0.31 vs. 0.09±0.03; movie: 0.04±0.15 vs. 0.02±0.01; neutral, closed: 0.43±0.14 vs. 0.42±0.16; open: −0.02±0.08 vs. −0.04±0.1; movie: −0.1±0.1 vs. −0.11±0.08). The amygdala was unaffected by this instruction (% signal change, negative, closed: 0.69±0.35 with task vs.0.7±0.2 without task; open: −0.18±0.13 vs. −0.18±0.04; movie: −0.32±0.14 vs. −0.31±0.16; neutral, closed: 0.2±0.2 vs. 0.2 0.1; open: −0.19±0.07 vs. −0.2±0.16; movie: −0.09±0.2 vs.−0.1±0.14).

**Figure 4:**
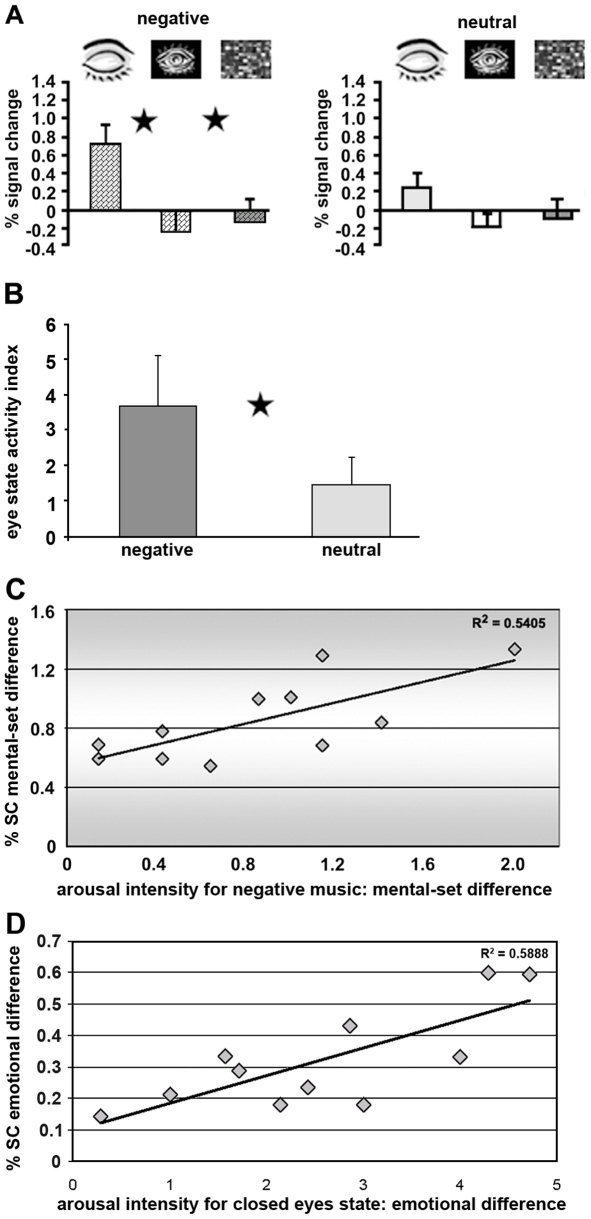
Sensitivity to emotional clips as reflected in activation profiles. (A) Average activation levels obtained in the amygdala. Stimulus type (x-axis) is indicated in the apertures above the graphs. The y-axis denotes an fMRI percent signal change relative to blanks. Asterisks denote a significant difference between eye states; *p<0.001*. Error bars, SEM. (B) ‘Index of eye state’ expressed by formula (close – open)/(close+open) indicates the ratios for negative and neutral stimuli. Asterisk denotes a significant difference between emotions; *p<0.05*. Error bars, SEM. (C) Relationship of the fMRI data and behavioral performance. Significant correlation (*p<0.05*) was found only for the amygdala between difference in measure of activation (negative closed – negative open, %SC – % signal change) and difference in the arousal intensity for negative clips. (D) Significant correlation (*p<0.05*) was found in the amygdala between difference in brain measures (negative closed – neutral closed, %SC – % signal change) and difference in the arousal intensity for eyes closed state.

To examine the magnitude of limbic areas' recruitment by the closed eyes position we performed a region of interest (ROI) analysis on our a-priori assumed core limbic area – the amygdala, and on spatially associated area – the anterior hippocampus (see Table S2 for Talairach coordinates). Figure 4A depicts the sampled activation level in the amygdala (based on internal localizer approach, see Materials and Methods) averaged across twelve subjects. An activation profile for the hippocampus was highly similar to that observed for the amygdala. As no main effect for hemisphere was found, we collapsed the data from the left and right hemispheres. A one-way ANOVA was performed within each music type (neutral, negative) with the three eyes position conditions (eyes closed, eyes open and scrambled movie) for the amygdala (F(1,11) = 6.0, *p = 0.001*) and hippocampus (F(1,11) = 22.9, *p<0.001*). Further, a post-hoc analysis (Fisher's PLSD) revealed a significant difference between the closed eyes position and the other two open eyes conditions for the negative music type both in the amygdala (*p<0.001* in tests: ‘open vs. closed’ and ‘scrambled-movie vs. closed’) and the anterior hippocampus (*p<0.001* in tests: ‘open vs. closed’ and ‘scrambled-movie vs. closed’). For the neutral music a significant difference between eye states was achieved in the anterior hippocampus (*p<0.001* in tests: ‘open vs. closed’ and ‘scrambled-movie vs. closed’) but not in the amygdala. Thus, as shown by the post-hoc analysis of the ROI data a tendency for a stronger interaction between eyes position and music type was present in the amygdala than in the hippocampus. To quantify this effect within the amygdala we calculated an index of eyes-state: (close – open)/(close+open) separately for negative and neutral music. The analysis revealed significant difference between the ratios for negative and neutral music (t(11) = 2.7, *p<0.05*, paired t-test) (Figure 4B). Together, as expected the whole brain and ROI analyses demonstrated a closed eyes effect in core limbic regions such as the amygdala and anterior hippocampus.

We then asked if the observed emotional selectivity of the closed eyes effect (i.e. ‘closed vs. open’ for negative music) in the amygdala corresponds to change in the emotional experience of these music clips. We investigated this possibility by correlating between the closed and open eyes differences in behavioral and brain measures in the amygdala separately for the negative and neutral music clips. Significant correlation was found only for the amygdala between difference in measure of activation (negative closed – negative open) and difference in the arousal intensity for negative (R^2^ = 0.5405, *p<0.05*, Figure 4C) but not neutral music clips (*p = 0.3*). This finding raises the possibility that the increased activation for negative music clips presented with eyes closed in the amygdala being related to a modified emotional experience as expressed by reports of increased arousing character of the music. Additionally, an expected effect of context was observed. Significant correlation was found in the amygdala between difference in brain measures (negative closed – neutral closed) and difference in the arousal intensity for eyes closed (R^2^ = 0.5888, *p<0.05*, Figure 4D) but not for eyes open state (*p = 0.5*).

In order to estimate the regional specificity of emotional modulation on eyes closed effect we performed a ROI analysis in three main foci of PFC activation obtained from eyes-state effect contrast: BA 47, BA 11 and BA 46/9 (see Figure 2 for distribution and Table S2 for Talairach coordinates). Again, no differences between hemispheres were found, thus the data were collapsed from both hemispheres. In the BA 47 a one-way ANOVA performed within each emotion revealed an effect (F(1,11) = 3.6, *p<0.05*) of eyes-state for both types of emotions - greater activation for closed eyes than open eyes for the negative as well as for the neutral emotions. Similarly, in the BA 11 the difference between closed and open eyes states was significant (*p<0.01*) for the negative and neutral emotions. In contrast, the BA 46/9 demonstrated a significantly (F(1,11) = 2.7, *p<0.05*) preferential activation for open eyes compared to closed eyes within negative and neutral emotion types. These findings demonstrated that within the PFC there is evidence for differential processing of the same music depending on the eyes state. Note that preference for emotion type was also opposite in these regions: while the BA 47 and BA 11 showed a preference for negative clips with eyes closed, the BA 46/9 demonstrated a preferential trend for neutral ones with eyes open (Figure 2).

### fMRI: Functional Connectivity

**Figure 5:**
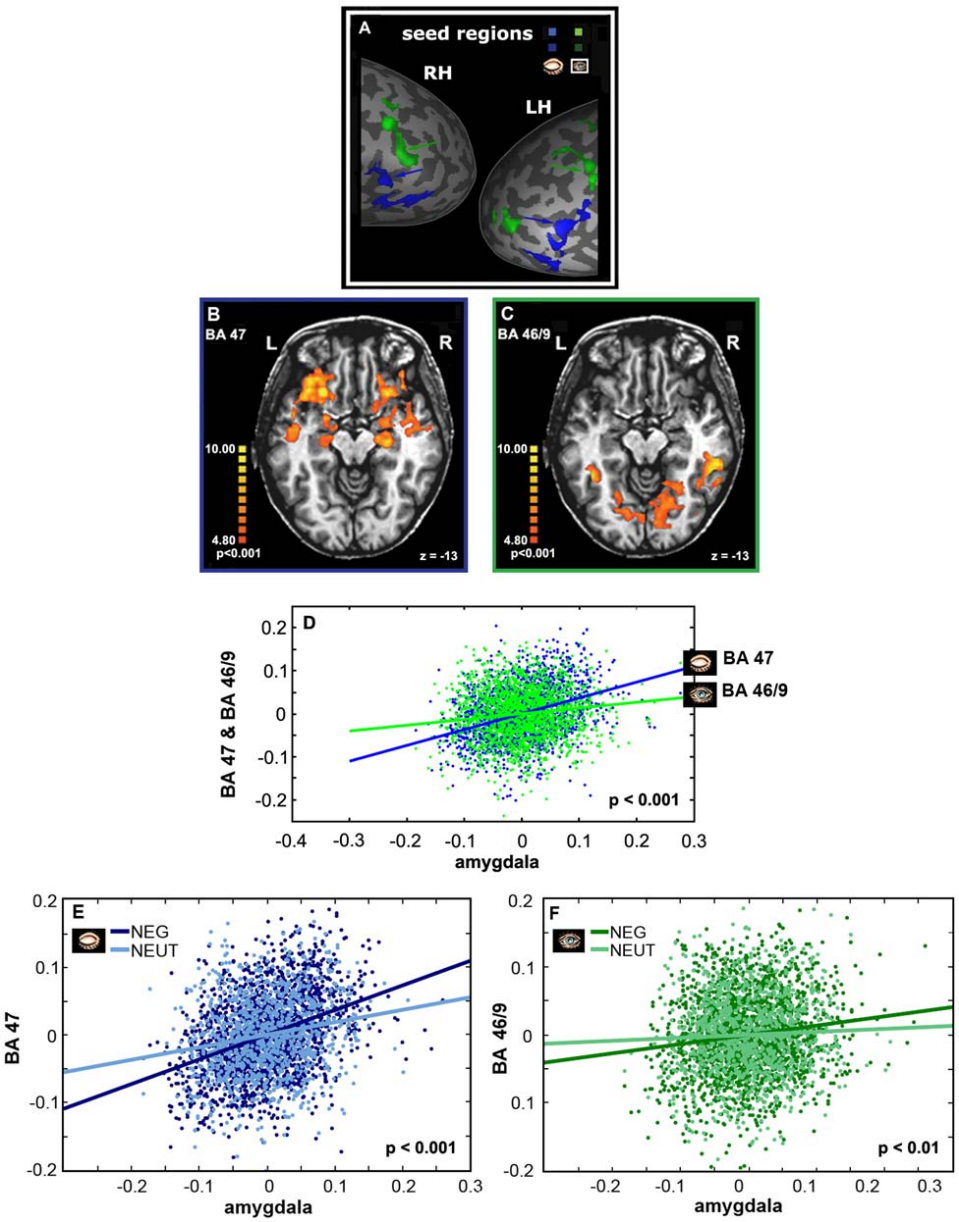
Correlation analysis for the PFC ROIs. (A) ‘Seed’ time courses defined separately for eyes open and closed are shown on the parts of unfolded hemispheres. ‘Seed’ time courses were used to compute a voxel-by-voxel fit for the (B) BA 47 and for the (C) BA 46/9. Note a strong connection of the amygdala to the BA 47. The color scale indicates significance level. R – right hemisphere, L – left hemisphere. (D) Correlated activity of the VPFC (BA 47) and amygdala in comparison to the correlated activity of the DLPFC (BA 46/9) and amygdala, corresponding to mental sets of eyes closed and open, respectively. Note a significant difference between correlation coefficients. (E–F) Co-activation analyses respectively to the emotional context in music. A statistical comparison between the co-activation in the negative and neutral conditions was performed for the BA47 (E) and BA 46/9 (F).

Having revealed an intriguing activity modification in the PFC and the amygdala depending on the mental set induced by eyes positions, we were interested in examining to what extent these effects can be explained by co-activation of these regions with other brain areas. To that end, we performed functional connectivity analysis with ‘seed’ time courses of preferential activation derived from the PFC ROIs (BA 47 for eyes closed, BA 46/9 for eyes open, Figure 5A) or the amygdala ROI (eyes closed with negative music). We then used these time courses as the General Linear Model (GLM) predictors to compute a voxel-by-voxel fit (see Materials and Methods for details), separately for each region. Fit evaluation revealed an intriguing effect: activation in the BA 47 was correlated with activation in the amygdala/anterior hippocampus complex and in the superior and middle temporal sulci and gyri (Figure 5B, *p<0.001*). The BA 11 also was tested for the eyes closed; the pattern of its connectivity was highly similar to that observed for the BA 47 (*p<0.05*, not shown). When the time course from the BA 46/9 was used as a predictor, activation was correlated mainly with distributed activation in the visual-related areas (Figure 5C, *p<0.001*). In order to quantify these mapping findings we conducted a direct statistical comparison between these two connectivity analyses. Figure 5Ddepicts that the correlated activity of the VPFC and amygdala was significantly stronger than the correlated activity of the DLPFC and amygdala, corresponding to mental sets of eyes closed and open, respectively (z score = 7.99, *p<0.001*). Then, to explore the link of these co-activation patterns to emotion in music we repeated the analysis with contrast time course obtained for each emotion separately, i.e. ‘seed’ time courses were defined once for the negative music and once for the neutral music (tests: ‘closed negative > open negative’, ‘open negative > closed negative’, ‘closed neutral > open neutral’, ‘open neutral > close neutral’). Statistical analyses of connectivity differences between eyes closed and eyes open states revealed significant differences for negative music (z score = 7.72, *p<0.001*) as well as for neutral music (z score = 3.4, *p<0.001*) for both VPFC and DLPFC. Moreover, a direct statistical comparison between the co-activation in the negative and neutral conditions was performed for the VPFC and DLPFC (Figures 5E–F). Although for both areas the co-activation was significantly stronger for negative music than neutral music, the correlation derived from the BA 47 was much stronger (z score = 5.18, *p<0.001* vs., z score = 2.44, *p<0.01*). The computed correlation coefficients for each subject and different conditions are provided in Table S3. These data demonstrate the consistency in the effect among subjects.

**Figure 6:**
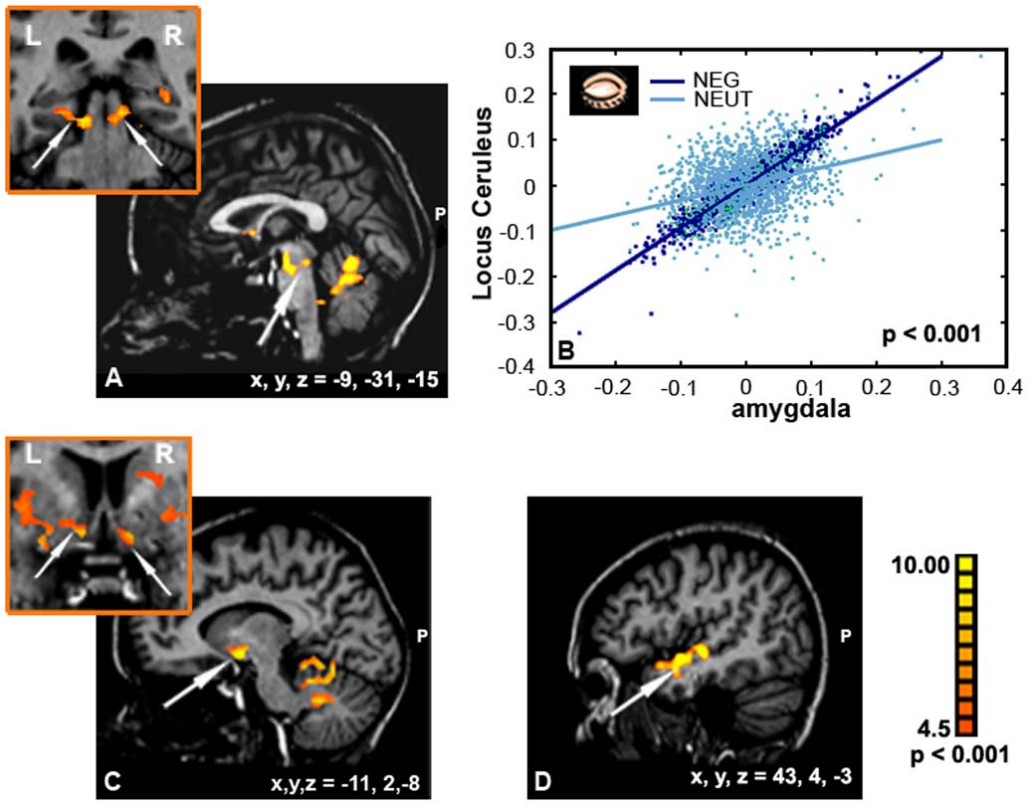
Correlation analysis for the amygdala ROI. An additional network of co-activation was revealed as during closed eyes in negative music. Arrows point to main regions of correlated activity with the ROI: (A) LC, (C) NAcc, and (D) anterior temporal gyrus. (B) Co-activation respectively to the emotional context is depicted. The correlated activity between the amygdala and LC during eyes closed was much stronger in the negative emotional condition than in the neutral condition.

The correlation analysis with ‘seed’ time course from the amygdala during eyes closed condition revealed an additional striking network of co-activation that was most prominent (*p<0.001*) in the bilateral pontine brainstem nuclei including the LC, ventral striatum areas including nucleus accumbens (NAcc) and anterior temporal gyrus region (Figure 6A, C, D). Again, we checked this co-activation respectively to the emotional context, using the amygdala once as a negative ‘seed’ obtained in the test ‘closed negative vs. open negative’ and once as a neutral ‘seed’ obtained in the test ‘closed neutral vs. open neutral’. As expected, statistical analyses revealed that the correlated activity between the amygdala and LC during eyes closed is much stronger in the negative emotional condition than in the neutral condition (z score = 39.73, *p<0.001*, Figure 6B).

Finally, to further quantify the connections between the PFC, amygdala and LC we applied the dynamic causal modeling (DCM) tool using SPM2. The DCM was aimed to examine two issues that were left open following the effective connectivity analysis. (i) Does the amygdala lead the enhanced inter-regional connectivity during the mental set of eyes closed? (ii) Is this enhanced connectivity modulated by emotionality in music and/or mental set of eyes closed?

**Figure 7:**
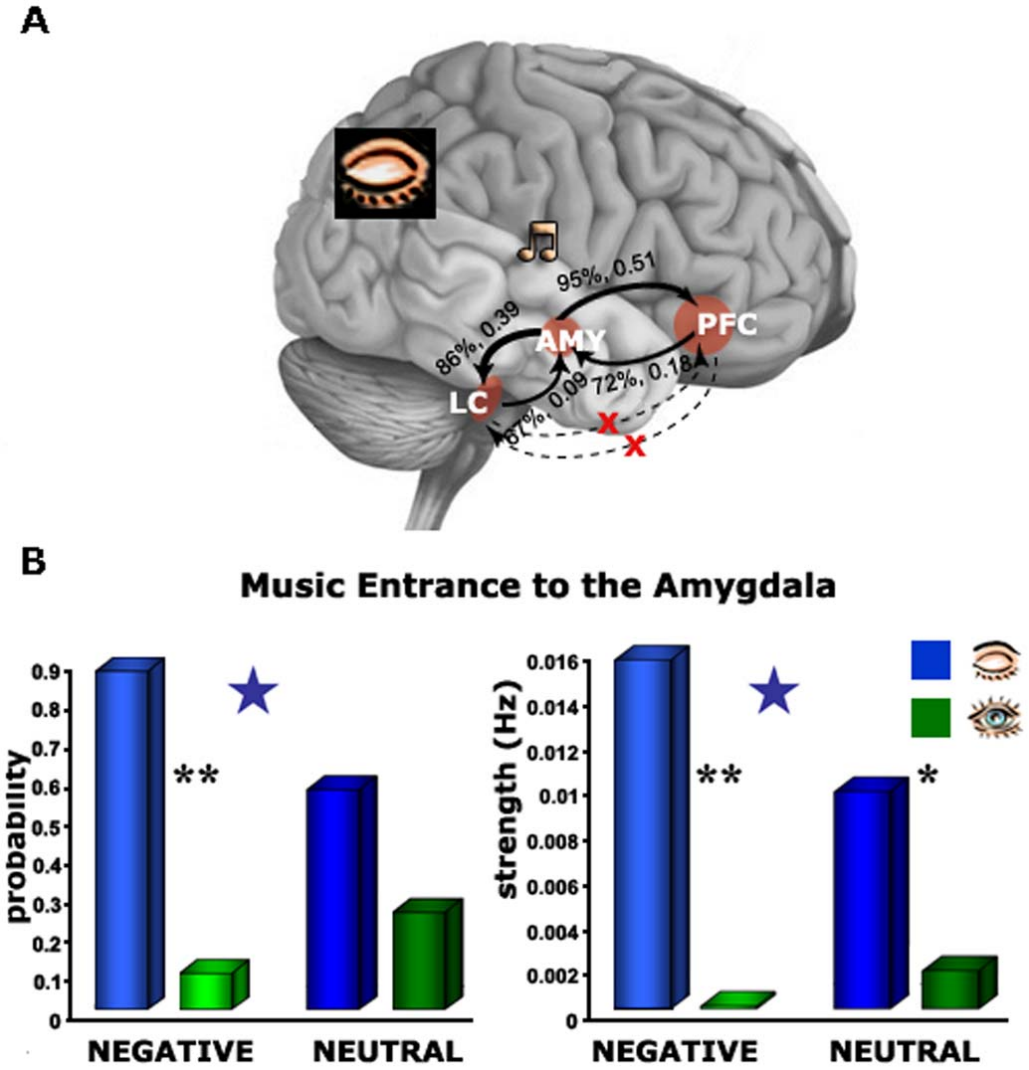
DCM – model and connectivity. (A) directions of the connectivity between the amygdala, PFC and LC during eyes closed. (B) The power of intrinsic connections indicated by probability and strength. Note the most probable and the strongest connection from the amygdala toward the PFC. *, *p<0.01*, **, *p<0.001*. Blue stars indicate a significant difference between the valences under closed eyes state (*p<0.05*).

The results for the first issue are presented on a brain model depicting the direction and strength of intrinsic connections by probability and intensity measures. Figure 7Ademonstrates that during the mental set of eyes closed and negative music the connectivity from the amygdala toward the PFC was more probable and stronger (95%, 0.51 Hz respectively) than from the PFC to the amygdala (72%, 0.18 Hz, respectively). Similarly, the connectivity from the amygdala to LC was more probable and stronger (86%, 0.39 Hz, respectively) than from the LC toward the amygdala (67%, 0.09 Hz, respectively). Paired t-tests confirmed the significance of these differences in connectivity between directions (*p<0.001*, for the amygdala-PFC vs. PFC-amygdala and the amygdala-LC vs. LC-amygdala). For the second issue we performed a two-ways ANOVA on measures of probability and strength for stimulus entrance (i.e. direct effect) to the amygdala, with regard to emotionality of music (i.e. negative, neutral) and mental set (i.e. closed, open). Figure 7Bshows that the eyes closed state led to high probability and strength of music entrance to the amygdala (F(1,13) = 16.45, *p = 0.001*), more so with negative than neutral valence (interaction values F(1,13) = 4.58, *p = 0.05*). Post-hoc analysis (Fisher's PLSD) revealed a significant difference between the eyes state under both negative (*p<0.001*) and neutral music (*p<0.01*) but there was a significant difference between the music types only under the closed eyes state (*p<0.05*). Finally, we examined whether the music itself modulates the inter-regional connections. This analysis revealed that music significantly modulates the connections of the amygdala to PFC (eyes closed: negative −0.589, neutral −0.622, eyes open: negative −0.613, neutral −0.537) and amygdala to LC (eyes closed: negative −0.577, neutral −0.605, eyes open: negative −0.559 neutral −0.545), albeit disregarding mental set or emotionality. All these effects were significantly greater than chance (*p<0.001*) but no significant difference was found between them. In other words, there was no evidence for selectivity in the modulation exerted by music on the connections between the amygdala toward the PFC and the LC.

### fMRI: Control Experiment

Closed eyes state is also associated with shutdown of visual input. This raised the possibility that the observed effect of eyes closed was driven by the corresponding effect of darkness. Our original experiment involved a condition of black screen during the eyes open state. Although this condition by itself did not produce the closed eyes effect seen in respect to the gray screen (see Figures 2, 3), it could still be claimed that it is a matter of magnitude and that black screen is not a good enough comparison for visual input shutdown. We tested this possibility in a control study where musical clips were presented in a complete darkness while subjects were asked to either open or close their eyes (see Materials and Methods). The whole brain analysis of this experiment revealed a similar closed eyes effect as our original experiment with strong preferential activation in the amygdala/anterior hippocampus complex, anterior temporal pole and VPFC for eyes closed (Figure S2 A). By sampling signals in the amygdala and the anterior hippocampus we could estimate a quantitative measure of this effect (Figure S2 B, an internal localizer approach was used, see Materials and Methods). In complete darkness, one would expect to have more activation in the amygdala in response to the negative music as compared the neutral music, independently of the eyes state. Indeed, relative to our original experiment, overall higher activation to music with eyes open was found (compare Figure S2 B and Figure 4A). Moreover, activation to neutral stimuli with eyes closed notably increased as compared to the original experiment. However, a central effect of significant prevalence of the amygdala and hippocampus activation with eyes closed over eyes open state remained for both negative (amygdala: (t(5) = 1.8, *p<0.05*; hippocampus: t(5) = 2.2, *p<0.05*) and neutral stimuli (amygdala: t(5) = 3.8, *p<0.05*; hippocampus: t(5) = 2.3, *p<0.05*). These results confirm that the overall closed eyes effect cannot be explained solely by the corresponding shutdown of visual input.

## Discussion

Our behavioral results demonstrated that closing the eyes enhances the unpleasant and arousing character of passively heard music excerpts. Although music did not vary, the experience of its emotionality differed following a mental set shift induced by moving from eyes open to eyes closed state. The neuroimaging results showed increases in activation with eyes closed in the amygdala, anterior hippocampus, temporal pole, and VPFC foci. Remarkably, only in the amygdala was this effect selective to music emotionality showing greater response to negative music with eyes closed than open. It can, therefore, be concluded that context-specific styles of attending can modify amygdala's activation in response to emotional music. Furthermore, correlation analysis with amygdala's activation when listening to negative music with eyes closed showed co-activation with the LC and the VPFC. This buttresses our assumption that, even in the absence of stimulus changes, a shift in mental set may affect emotional perception by parallel recruitment of distributed neural operations related to ‘low-visceral’ as well as ‘high-appraisal’ related processing, respectively. Here, we discuss how such amygdala driven inter-level neural recruitment might affect the subjective emotional experience.

### The Effect of Eyes Closed on Amygdala Activation

Although behaviorally emotional music excerpts were rated as more negative and arousing than neutral sound excerpts in both open and closed eyes states, selectivity to emotionality in the amygdala was shown only in the latter (Figures 3–4). The low sensitivity of the amygdala to the emotional value associated with music presented with eyes open is in line with prior PET and fMRI studies in humans [22], [23], [27]. Though opposite evidence for sensitivity of the amygdala to emotional music with eyes open has also been presented especially with regard to the semantic structure of music [24], [25]. These differences possibly arise from the nature of stimuli that have been used in studies. The dissonant or scrambled sounds used by Blood et al. [22] and Levitin and Menon [23] may have evoked less unlikable feelings than unpleasant real music excerpts in Koelsch et al. [24], [25].

The amygdala's critical role in establishing the emotional context of sounds is suggested by lesion studies using auditory fear conditioning in rodents [28], and by studies in patients with amygdala excisions investigating the effect of scary music or unpleasantness of dissonance in music [29], [30]. Further support for the sensitivity of the amygdala to emotional context comes from few imaging studies using various parameters of fear conditioning paradigms, meaning of sounds such as crying, and accompanied unpleasant narrative via words or films [26], [31]–[33]. Here we show that the amygdala is sensitive to context change derived from shifting mental set without changing the stimulus complexity. This further supports our prior interpretation for increased amygdala activity to music when combined with films, as related to modified processing style (i.e. mental set) rather than changing mode of stimulation (i.e. concrete movie and music vs. abstract music alone) [26]. As previously conjectured, in the presence of negative music, closing the eyes may be associated with increases in attention to visceral cues of emotions, as well as appraisal and conceptual processes. To further examine the way the amygdala is mediating such processes we looked at its co-activation with regions most typically proposed to be involved in their operation: the LC and VPFC, respectively.

### The Effect of Eyes Closed on Amygdala's Connectivity with the LC

The finding that the amygdala significantly co-activated with brainstem noradrenergic nuclei such as the LC is consistent with animal studies showing dense reciprocal connections between the amygdala and these brainstem nuclei [34]. This connection was suggested to serve enhanced detection of threat signals through vigilance, alertness, and orientation processes [20], [35]. In accordance, a recent fMRI study demonstrated increased correlated activation between the amygdala and the LC when remembering faces that had been encoded in an emotional negative rather than neutral context [36]. Intriguingly, another imaging study found that unaware fear signals were more likely to elicit activity in the LC than aware fear signals [37]. This sort of finding supports the idea that the LC serves a relatively low level ‘alarm system’ that incorporates alerting and orienting signaling to threat-related stimuli even without full awareness of them. Our current findings suggest that the amygdala's connections to adrenergic brainstem nuclei may mobilize such a low level system under perceptual context which calls for higher alert (e.g. diminished external visual information). Though, uniquely here we show that such low level operation is recruited even for processing relatively complex cultural based emotional stimuli such as music.

In addition to evidence of increased co-activation between the amygdala and LC when subjects listened to negative music with eyes closed, our causal analysis revealed that this inter-regional connection was more strongly driven by the amygdala than by the LC. The LC has been indicated as enabling an appropriate electrodermal reactivity (i.e. visceral cues of emotions) in the face of threat, therefore its recruitment by the amygdala under condition of increased emotionality support a neural basis for the ‘somatic marker theory’ [38]. However, we did not find valence selectivity in the modulation effect on this inter-regional connection under the eyes closed condition suggesting that the emotional value is not determined by the strength of these connections. Taken together, our findings propose a picture whereby closing the eyes in the presence of negative music is associated with arousal/vigilance modulations exerted through enhanced amygdala's drive of brainstem nuclei including the LC. The LC-amygdala back-projection revealed by the causal analysis suggests a low level modulation of emotional processing, wherein brainstem nuclei would facilitate the orienting to affective signals possibly by providing the amygdala visceral cues of emotion [13]. Interestingly, the LC did not show increased overall activity to negative music with eyes closed, consistent with evidence that arousal modulation may increase effective connectivity between the LC and attention-related cortical regions without increasing activity within these regions [20], [35].

### The Effect of Eyes Closed on Amygdala's Connectivity with the PFC

It was suggested that the amygdala is critical to both immediate signaling of threat in the environment and to slower concept based cognition regarding negative consequences of such threat [39]. Our findings support a possibility that the latter is carried out via amygdala's connection to the VPFC. The applied causal analysis suggests that the enhanced effective connectivity between the amygdala and VPFC is driven by the former when passively perceiving an emotional stimulus, as in our study (Figure 7).

It was previously claimed that the VPFC is involved in appraisal-related mechanisms [40]–[42]. In contrast to the amygdala, the VPFC foci of the closed eyes effect did not show significant selectivity to the emotional content of music (Figure 2, bar graphs). The weak selectivity to emotional valence of this region has been suggested by animal and human lesion studies, respectively showing that the PFC is not critical for acquiring a fear conditioned response [43] and for the association formation of stimuli with a negative meaning [44].

However, it is yet possible that the PFC may show selectivity when appraisal of emotional stimulus is driven by actively directing focus of attention through task demands. Indeed, we found that the eyes closed VPFC foci showed a slight trend toward greater selectivity to emotion when subgroup of our subjects were instructed to judge the emotionality of musical clips, while the amygdala was unaffected by this instruction (see Results). This fits with the proposed role of the VPFC in the regulation of emotional experience via appraisal mechanisms [45] and affective decision [44], [46]. Moreover, the results from our causal analysis are consistent with suggestions that the emotional sensitivity of the VPFC depends on the modulation of its afferents from the amygdala [47]. However, possibly by manipulating appraisal directly the leading direction of the PFC-amygdala connection might have changed. Indeed, it has been shown that the appraisal of the emotional valence of stimuli is associated with modulations of functional connectivity between the amygdala and the VPFC [48], and that this functional coupling is diminished in patients with depression relative to healthy controls during re-appraisal of negative stimuli [45].

While our results did not highlight any direct connection between the LC and the PFC, the former could indirectly modulate the processing of vigilance-related visceral cues in the PFC either through its effect on the amygdala or through striato-cortical loops [49]. Indeed, the ventral striatum receives glutamatergic afferent input from brain regions that have been associated with task execution with eyes closed in our study, including the amygdala, hippocampus, and VPFC. Furthermore, this interpretation is consistent with the co-activation observed between the amygdala and the ventral striatum in the whole brain effective correlation analysis (see Figure 6) indicating a possible effect of eyes closed on gating mechanisms selective to emotion value in music [50].

### Segregated PFC Activation with Relation to Mental Set Operations

The close relationship of the VPFC but not DLPFC activation foci with the amygdala (see Figure 5) is consistent with a proposed segregation of VPFC and DLPFC functional systems in monkeys [12], [42]. The VPFC which includes the eyes closed activation in our study (i.e. BA 47 and BA 11) is reciprocally connected with the amygdala, hippocampus and anterior inferior temporal area and was proposed to be involved in multimodal internally driven processing [12], [47]. On the other hand, the DLPFC, which includes the eyes open activation patches in our study, is closely connected to the low- and high-order visual areas and superior temporal sulcus (STS) region and was involved in processing stimuli based on the externally determined features [40]–[42]. This dissociation in brain activation pattern related to eyes position is in line with a study by Hariri et al. [51] that used an emotional paradigm in the visual domain. The authors showed that an implicit emotional processing task was associated with increased activity in the amygdala/hippocampal region. In contrast, an explicit emotion processing task requiring the interpretation of emotions based on acquired knowledge and linking them to linguistic labels was associated with increased activation in the DLPFC. Based on these findings we can interpret our results suggesting that in the eyes closed condition when all visual inputs become unavailable, compensatory mechanisms for processing non-visual information from the environment - in this case mediated by the ventral system - may be automatically recruited. The VPFC was postulated as important for the identification of emotional significance of a cue and for the regulation of current affective states. In contrast, the dorsal system known to be important in explicit cognitive processes is active during the open-eyes state when all mechanisms could be involved in the high-order cognitive tasks (i.e. planning, searching, etc.) [52]. Our finding reminds another study by Baumgartner et al. [53], which compared emotional pictures presented either alone or combined with congruent emotional musical excerpts. Combining emotional music with the pictures enhanced their emotional significance alongside with increased activation in a ventral neural system comprising the amygdala, brainstem nuclei and ventral regions of the anterior cingulate cortex and PFC.

### Potential Perception-related Confounds

Several perception-related processes could contribute to our findings. For example, could enhanced emotional experience be related to intensive imagery induced by the closed eye position? Our behavioral results make this possibility very unlikely since they did not reveal a change in concreteness of the sensory experience while closing the eyes (Figure 1D). Brain wise, the closed eyes state did not evoke significant increase in activation in exclusively visual areas as would be expected if the imagery was object selective [54], [55]. Also no visually-related areas were revealed by correlation analyses for the eyes closed foci in the VPFC or the amygdala with whole brain activation (Figure 5). However, increased activation was seen for eyes closed in the multi-modal regions, such as the STS and the inferior parietal sulcus (IPS) that were implicated in mental imagery by others [56]. Though this finding is consistent with some form of imagery, its exact meaning is unclear. Another interpretation could be that the eyes closed position modifies mental schemes related to spatial attention and self-body reference, both shown to be mediated by the STS and IPS [57]–[59]. Further research should explore if and how this perceptual modification can change emotional experience of music.

Another perceptual effect could be related to visual input withdrawal with eyes closed. This possibility was tested in the original experiment by an open eyes condition where subjects stared at a black screen while listening to the music clips. The lack of observable effect of negative music on the amygdala during this condition suggests that the closed eyes effect on the emotional experience of music cannot be explained by low level visual input during eyes closed (see Figure 3). However, in an additional control study, where subjects opened their eyes in complete darkness, the magnitude of the emotional selectivity of eyes closed effect in the amygdala seemed weaker (see Figure S2 B in comparison to Figure 4A). A potential confounding factor was represented by the fact that half of the subjects in the control study were repeating the experiment, thus potentially dampening or otherwise changing the emotional response to the music. This possibility needs to be tested in greater depth in future studies using corresponding physiological measures of arousal.

Finally, two additional confounds need to be addressed. First, it could be argued that subjects found the experience of lying in a scanner listening to music with eyes closed particularly novel thus resulting in differences in the amygdala [60]. Our brain results argue against this likelihood. We base this argument on the fact that the eyes closed effect was selective for negative music, whereas subjects experienced neutral music with eyes closed as well. Second, it could be suggested that a contributing factor to the closed eyes effects could be related to the lack of gaze control when eyes are closed. This suggestion seems to be somewhat unlikely in our study because all conditions with eyes open were presented under the free viewing and no fixation point was added.

In summary, our findings support a system-based model in which context-related variations of processing style induced by closing the eyes can modify individuals' emotional experience of negative stimuli via modified amygdala's activity. Furthermore, the amygdala's connectivity with the LC and VPFC seems to contribute to such modification, by selectively recruiting, respectively brain operations related to low level processing of visceral cues and/or high-level cognitive based processing of emotions. Our secondary causal analysis further points to the primacy of the amygdala in driving possible interplays between these levels of operation under unguided perceived emotionality in music. The fact that the amygdala is co-operated with regions involved in both low and high levels of emotional processing supports the integrative view of the ‘conceptual act model’ in explaining individual diversities in emotional perception of stimuli. Speculatively, it can be suggested that disturbed emotional experiences such as depression and anxiety are evoked by imbalanced co-operation of these paths of the emotional system. Future electrophysiological studies can explore whether these amygdala's connections are characterized by specific activation features such as neural synchronization at characteristic frequencies.

## Materials and Methods

### Subjects

Twenty six healthy subjects participated in the study. Eleven subjects (5 females, ages 25–41 years) took part in the pre-scan behavioral measurements. Twelve subjects (6 females, ages 28–36 years) participated in the original fMRI experiment. Six subjects (4 females, ages 23–36 years) participated in the control experiment (3 of them had also taken part in the original experiment). None of the subjects had been musical expert; 6 subjects had basic musical education in youth but did not practice to play during last 15 (and more) years. All subjects had normal hearing and provided written informed consent. The IRB committee of the Tel-Aviv Sourasky Medical Center approved the experimental protocol.

### Stimuli

Overall, the music clips consisted of 28 clips, 12 s each. The clips were un-familiar to the subjects. They contained no human voices or specific object-related sounds (e.g., a clock ticking, footsteps, etc.). Initially, the music clips were characterized as positive (joyful; used in the pre-scan behavioral session only), negative (scary), or neutral (for examples, listen to Stimuli S1, S2, S3, S4, S5, S6, S7 and S8). The positive clips were recorded from commercially available CDs and film sound tracks. The negative clips were composed (cut and edited) from a horror film sound tracks, from music pieces (using Wavelab 4.0 from Steinberg, Steinberg Media Technologies, GmbH, Hamburg, Germany), or created in-house using music software (Cubase VST 5 from Steinberg, Reaktor 3.0 and Kontakt 1.0 from Native Instruments Software Synthesis, GmbH, Berlin, Germany). The neutral clips were recorded from commercially available CDs or created in-house using music software (Cubase VST 5 from Steinberg, Reaktor 3.0 and Kontakt 1.0 from Native Instruments Software Synthesis, GmbH, Berlin, Germany). Finally, the emotional value of the clips was defined based on a pre-scanning behavioral rating (below).

The average decibel level of all music clips was equalized using Steinberg Wavelab 4.0. Importantly, all stimuli had similar acoustical characteristics (all major-minor tonal music), melodic contour, rhythmic structures and tempo, so, it is highly unlikely that simply the bottom-up processing of the stimulus characteristics contributes to activation patterns when contrasting different conditions. Also, there were no unexpected events in the negative clips preventing the possibility that the amygdala may be triggered by them more effectively than by the neutral clips.

The video clips were collected from commercial films. They were chosen according to the following requirements: (i) clips were emotionally neutral; (ii) contained no dialogue, and (iii) showed no widely familiar actors or scenes. The clips have been converted into the black and white ones and each frame was scrambled using the Matlab programs. The scrambled clips were too difficult to follow the movie due to very short presentation of each frame (40 ms long). Importantly, coupled with emotional music, video clips could be interpreted as both negative and neutral.

### Pre-scan Behavioral Tests of Stimuli

Two pre-scan behavioral sessions were conducted aiming to evaluate the emotional quality of the musical clips intended for the fMRI paradigm. The valence levels of musical clips (negative/neutral/positive) were checked in the first session by presenting 11 naive subjects who did not intend to participate in the fMRI experiments with 28 different natural musical clips (each 12 s long and separated by intervals of quiet blank periods). These subjects were required to estimate the valence (on a scale from −5 to 5) and arousal (on a scale from 0 to 10) levels of the clips. Ratings for each clip were averaged across subjects and were characterized as either neutral (from −1 to 1), positive (from 1 to 5) or negative (from −1 to −5). All positive clips and the three negative clips defined as outliers (i.e., more than 2 STDs away from the mean rating for negative clips) were excluded from the clip pool. Two weeks later, the 18 selected clips (9 negative with an average valence of −2.1±0.5 and 9 neutral with an average valence of 0.6±0.4) were presented together with scrambled video clips and re-evaluated in a similar manner. These results were also averaged across subjects and four outlier clips were excluded from the final pool of stimuli.

### Experimental Design

#### Original fMRI experiment

Overall, the experiment was composed of 42 blocks. Specifically, 14 finally pre-selected clips (7 negative and 7 neutral, examples: Stimuli S1, S2, S3, S4, S5, S6, S7 and S8) were presented to the subjects in 3 different states: eyes open with a black screen, eyes open with a scrambled video and eyes closed (Figure 1A). Importantly, all open eyes conditions were presented under the free viewing - no fixation point was presented on the screen. To avoid priming effects, the presentation of negative and neutral clips in relation to eyes position was equally distributed throughout the experiment. Each clip was 12 s long and alternated with 6 s blank periods. The experiment lasted a total of 846 s. The paradigm started with a 15 s black screen blank period and ended with a similar 12 s period. Verbal instructions to open or close eyes lasted 3 s each. Six subjects in the original experiment were requested to covertly perform the valence-rating of each clip immediately after its presentation (on a scale from −5 to 5; no arousal and abstract level were rated) and six subjects were scanned while passively listening to music.

#### Across-subject arrangement

Two order sequences of the paradigm were created. Although the versions consisted of the exactly the same set of musical and video clips, the order of specific eye position+music coupling was different across subjects.

#### Control fMRI experiment

This study was aimed to control for the possibility that the difference between eyes closed and eyes open is due to change in the level of darkness. The experimental design was identical to the original experiment with the exception that musical clips (in auditory conditions only, without video) were presented in complete darkness. Subjects listened to neutral and negative music with eyes open looking at a black screen, and also listened to it with eyes closed. Importantly, video clips were not excluded from this experiment – stimulation was manipulated in such a way that the projector's bore was open just before the condition containing video stimulation and closed immediately after it.

### Behavioral Experiment

Behavioral measurements were conducted outside the magnet on all subjects who participated in the original fMRI experiment several weeks later. The delay was inspired by several motivations: (i) we did not measure behavioral performance during the experiment in order to minimize possible cognitive demand, which would impact amygdala function as well; (ii) immediate rating after the scan was not carried out because of subject's possible lower sensitivity to stimuli after their repeated presentation in the magnet. Subjects were presented with the same stimuli that were used during the scan. The blank periods were extended to 15 s to allow enough time for their response. The subjects were required to score the emotional valence (−5 to 5), the arousal feeling (0 to 10) and the abstract level (0 to 10) of all the stimuli. To ensure that effect of shifting processing style is not related to increase in imagination, we estimated abstract level of the stimuli. Subjects were asked to evaluate at what degree they imagine (if so) the concrete objects or scenes while listening to music. The average rating was calculated per condition within and across subjects (Figure 1B–D).

### MRI Acquisition

Subjects were scanned in a 1.5 T Signa Horizon LX 8.25 GE scanner (original experiment) and in a 3 T G3 GE scanner (Milwaukee, WI, USA) (control experiment) with standard birdcage head coils. The scanned volume included 25–27 nearly axial slices of 4 mm thickness and 0 mm gap and covered the entire cortex. Blood oxygenation level dependent (BOLD) contrast was obtained with gradient-echo echo-planar imaging (EPI) sequence (TR = 3000 ms, TE = 55/35, flip angle = 90°, field of view 24×24 cm^2^).

A whole-brain spoiled gradient (SPGR) sequence was acquired on each subject to allow accurate cortical segmentation, reconstruction and volume-based statistical analysis. T1-weighted high resolution (1.1×1.1 mm^2^) anatomical images (124 images, 1.2 mm thickness) of the same orientation as the EPI slices were acquired to facilitate the incorporation of the functional data into the 3D Talairach space [61].

Stimuli were generated on a PC. During scanning, the visual stimuli were presented to the subjects via an LCD projector (Epson MP 7200). Subjects viewed them in a titled (∼45°) mirror positioned over their foreheads. The audio stimuli were presented via the headphones.

### Data Analysis

fMRI data were analyzed with the BrainVoyager software package (Goebel, 2000, Brain Innovation, Maastricht, The Netherlands) and with complementary in-house software. The first three images of each functional scan were discarded. The functional images were superimposed on 2D anatomical images and incorporated into the 3D data sets through trilinear interpolation. The complete data set was transformed into Talairach space [61]. Pre-processing of functional scans included 3D-motion correction, linear trend removal, slice scan time correction, and filtering out of low frequencies up to 3 cycles/experiment. The cortical surface of each subject was reconstructed from the 3D SPGR scan. The procedure included segmentation of the white matter using a grow-region function, the smooth covering of a sphere around the segmented region, and the expansion of the reconstructed white matter into the grey matter. The surface of each hemisphere was then unfolded, cut along the calcarine sulcus and flattened.

### Statistical Analysis

#### Single subject analysis

GLM [62] statistics were used. Throughout the statistical analysis, a hemodynamic lag of 3–6 s was assumed for the model of each subject by maximizing the extent of the overall activations. A box-car predictor was constructed for each experimental condition except the blank period, and the model was independently fitted to the signal of each voxel. A coefficient was calculated for each predictor using a least-squares algorithm.

#### Multi-subject analysis

A multi-subject analysis was also performed. The time series of images of all subjects were converted into Talairach space and z-normalized. For each subject, the relative contribution of the predictors for each contrast was estimated separately and then the significance at the multi-subject level was calculated from the obtained set of values. Computation of significance values in the activation maps was based on the individual voxel significance and on the minimum cluster size of 10 voxels [63]. The multi-subject maps were obtained using a random effect procedure [64] and projected on a single flattened Talairach normalized brain. Statistical levels were indicated by the color scales. ANOVA was calculated via StatView 5.0.1 software.

#### Internal localizer approach

Details of the approach were previously published [65]. Briefly, one subset of the repetitions of a condition was used to localize the ROI (biased statistically as a part of the test), while a signal in the complementary subset of the repetitions (unbiased statistically) was used to evaluate the activation level. Specifically, two statistical tests were conducted for each localizer test (e.g. ‘open eyes’ vs. blank period). In each test, four repetitions of the condition were biased statistically while the other repetitions of this condition were unbiased statistically, and activation levels during these blocks were measured separately. There was no overlap between the unbiased repetitions of the two tests. Similar activation profiles in the biased and unbiased subsets can be used as an indication of a high signal-to-noise ratio. The levels of activation were analyzed separately for the biased and unbiased data: they were first averaged for each subject and then averaged across subjects.

#### Correlation maps analysis

The correlation analysis was based on ‘seed’ time courses [66]–[69]. ‘Seed’ ROIs were anatomically defined for each subject in each cortical hemisphere as a cluster with the highest activation level. Average ‘seed’ time courses were obtained for each subject by averaging the time series of all voxels in the specific ROI (areas BA 46/9 and BA 47, for example). These average time courses were used as a GLM predictor to compute a voxel-by-voxel fit (analogous to linear correlation). Since consecutive fMRI data points of the regressor are not statistically independent due to the nature of the hemodynamic response, the fit was evaluated after removing the auto-regression factor (AR1 model). A second-level random-effect analysis was applied to determine the brain areas that showed significant functional activity across subjects. In contrast to the normally applied procedure in which every subject's dataset is fitted with the same design matrix, we used a different design matrix for each subject, based on the subject's actual data (‘seed’ time courses from the same ROIs) so that the final map reflects regions whose activity is correlated to the activity in the same ‘seed’ location across subjects.

#### DCM

For the purpose of DCM, fMRI data were reanalyzed using Statistical Parametric Mapping software package, SPM2 (Wellcome Department of Imaging Neuroscience, London, UK). DCM is a nonlinear systems identification procedure that uses Bayesian rules to estimate the influences that one neural system exerts over another and how this is affected by the experimental context [70], [71].

DCM analysis was performed on 14 subjects: 11 out of 12 subjects participated in the original fMRI experiment (one subject was eliminated due to technical problems) and 3 subjects participated in the control fMRI experiment but did not participate in the original experiment (the data sets of 3 subjects participated in both experiments were taken from the original experiment). Preprocessing of functional images included motion correction (realignment to the first volume), slice time correction (to the middle slice) and normalization to the standard EPI template of the Montreal Neurological Institute (MNI).

Statistical analysis was relied on a GLM. Events were time-locked to onset of stimulus presentation and regressors modeling stimulus events were convolved with a canonical hemodynamic response function. T-statistical maps were obtained by contrasting hemodynamic responses during epochs of stimuli presentation of negative music and neutral music with eyes closed. The analysis of individual subjects was performed at a significance threshold of *p<0.05* (uncorrected).

The statistical maps of the negative vs. neutral music with eyes closed contrast were used to define volumes of interest (VOI) for each subject. The voxel of maximum activation within each VOI was served as a center of spherical volume (PFC – 6 mm, amygdala - 3 mm, LC – 2 mm) and defined by the volume of interest tool integrated in SPM2 (see Table S1 for details on VOIs).

For each subject a model was defined: an input to the amygdala, and values of intrinsic connectivity in each direction were extracted and served for a 2-way ANOVA (factors: valence-negative, neutral; eyes-closed, open). Probability and strength of stimuli entering the amygdala were extracted and evaluated in a t-test (probability – more than 50%, strength – in Hz). Modulating effects of the negative music and neutral music with eyes closed were modeled on connections of the amygdala and LC and connections of the amygdala and PFC.

## Supporting Information

Figure S1 Activation maps of limbic regions in single subjects. Activation patterns revealed during eyes closed stimulation (eyes closed > blank) in the amygdala/anterior hippocampus region for six different subjects. Compare a dramatic emotion-related effect revealed for the negative and neutral clips. Regions of interest are marked by circles. The color scale indicates significance level. L - left hemisphere, R - right hemisphere, COR - coronal, TRA - transversal.
(6.99 MB TIF)

Figure S2 Control experiment - complete darkness effect. (A) Multi-subject activation patterns (N = 6) obtained by the contrast ‘eyes closed > eyes open’ in the control study are shown on the transversal view. A strong preferential activation for eyes closed was found in the amygdala and the anterior hippocampus. The color scale indicates significance level. L - left hemisphere, R - right hemisphere, TRA - transversal. (B) Average activation profiles were obtained in the amygdala (left) and the anterior hippocampus (right) in the ‘eyes closed > eyes open’ test. Apertures above the graphs specify stimuli type. The y-axis denotes an fMRI percent signal change relative to blanks. Note a significant effect in the amygdala/anterior hippocampus for the eyes closed state. The asterisk denotes a significant difference between the eyes open and eyes closed states (p<0.05). Error bars, SEM.
(5.80 MB TIF)

Table S1 Volumes of interest used in DCM. Size and location of VOI. The location is given as MNI coordinates. The voxel of maximum activation within each VOI was served as a center of spherical volume.
(0.10 MB DOC)

Table S2 Regions of interest used in functional analysis. Size and location of regions of interest. The location is given as Talairach coordinates of the center of the region (X, Y, Z).
(0.03 MB DOC)

Table S3 Correlation coefficients. Correlation coefficients for each subject are presented for un-normalized time-courses. Co-activation of the amygdala with area BA 47 and co-activation of the amygdala with area BA 46/9 were computed. Activation in the BA 47 was obtained in contrast ‘close > open’ for the negative and neutral stimuli separately. Activation in the BA 46/9 was obtained in contrast ‘open > close’ for the negative and neutral stimuli separately.
(0.05 MB DOC)

Stimulus S1 Negative Stimulus 1
(1.06 MB AVI)

Stimulus S2 Negative Stimulus 2
(1.06 MB AVI)

Stimulus S3 Negative Stimulus 3
(1.06 MB AVI)

Stimulus S4 Negative Stimulus 4
(1.06 MB AVI)

Stimulus S5 Neutral Stimulus 1
(1.07 MB AVI)

Stimulus S6 Neutral Stimulus 2
(1.06 MB AVI)

Stimulus S7 Neutral Stimulus 3
(1.06 MB AVI)

Stimulus S8 Neutral Stimulus 4
(1.06 MB AVI)
