# Supplementary figures and images for: Eyes Wide Shut: Amygdala Mediates Eyes-Closed Effect on Emotional Experience with Music
Source: PLoS One. 2009 Jul 15;4(7):e6230. doi: 10.1371/journal.pone.0006230 (PMC2705682; doi:10.1371/journal.pone.0006230)

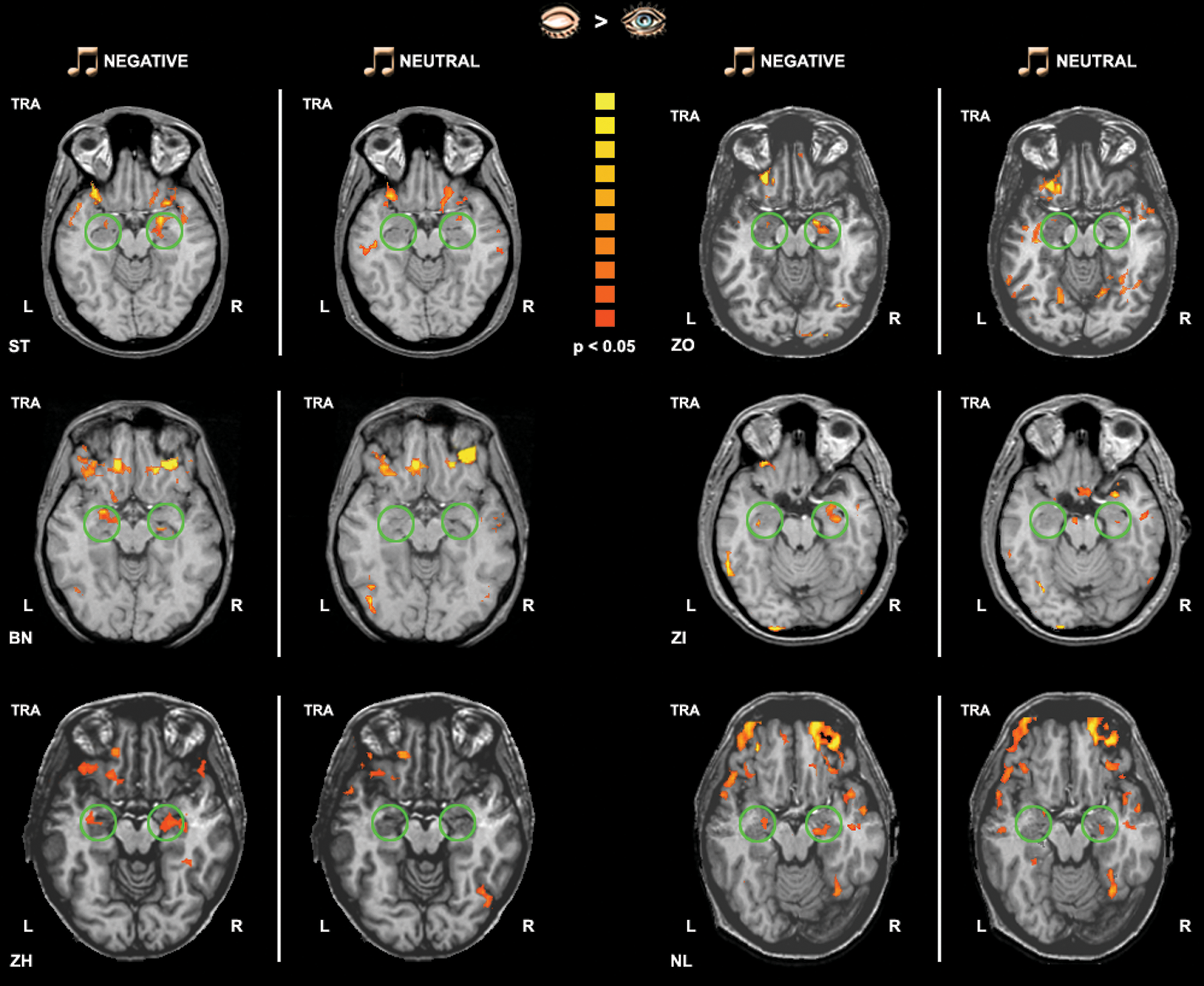

Supplement: Figure S1 — Activation maps of limbic regions in single subjects. Activation patterns revealed during eyes closed stimulation (eyes closed > blank) in the amygdala/anterior hippocampus region for six different subjects. Compare a dramatic emotion-related effect revealed for the negative and neutral clips. Regions of interest are marked by circles. The color scale indicates significance level. L - left hemisphere, R - right hemisphere, COR - coronal, TRA - transversal. (6.99 MB TIF) [file pone.0006230.s001.tif]

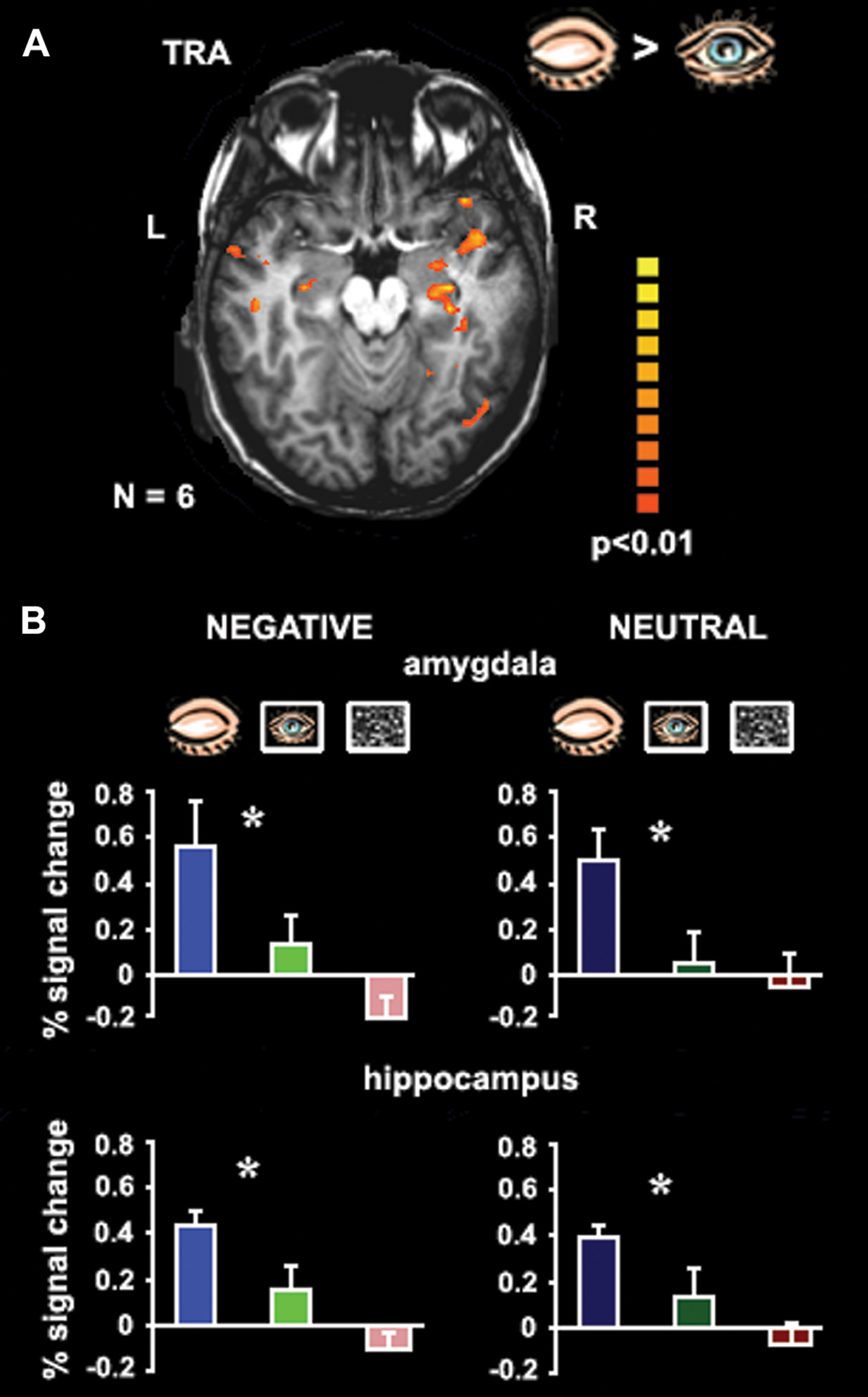

Supplement: Figure S2 — Control experiment - complete darkness effect. (A) Multi-subject activation patterns (N = 6) obtained by the contrast ‘eyes closed > eyes open’ in the control study are shown on the transversal view. A strong preferential activation for eyes closed was found in the amygdala and the anterior hippocampus. The color scale indicates significance level. L - left hemisphere, R - right hemisphere, TRA - transversal. (B) Average activation profiles were obtained in the amygdala (left) and the anterior hippocampus (right) in the ‘eyes closed > eyes open’ test. Apertures above the graphs specify stimuli type. The y-axis denotes an fMRI percent signal change relative to blanks. Note a significant effect in the amygdala/anterior hippocampus for the eyes closed state. The asterisk denotes a significant difference between the eyes open and eyes closed states (p<0.05). Error bars, SEM. (5.80 MB TIF) [file pone.0006230.s002.tif]
