# Supplementary material for: Eyes Wide Shut: Amygdala Mediates Eyes-Closed Effect on Emotional Experience with Music
Source: PLoS One. 2009 Jul 15;4(7):e6230. doi: 10.1371/journal.pone.0006230 (PMC2705682; doi:10.1371/journal.pone.0006230)
Supplement: Table S1 — Volumes of interest used in DCM. Size and location of VOI. The location is given as MNI coordinates. The voxel of maximum activation within each VOI was served as a center of spherical volume. (0.10 MB DOC) [file pone.0006230.s003.doc]

**Supplementary Table 1.** *Volumes of interest used in DCM*.

| **Cortical Region** | **Subjects** | **MNI coordinates** | | | | | | | | |
| --- | --- | --- | --- | --- | --- | --- | --- | --- | --- | --- |
|  |  |  |  | **LH** |  |  |  | **RH** |  |  |
|  |  | **x** | **y** | **z** | **Cluster size** |  | **x** | **y** | **z** | **Cluster size** |
| **Amygdala** | BN | -16 | -4 | -16 | 6 |  | 16 | -8 | -16 | 5 |
|  | EO | -26 | -10 | -20 | 14 |  | 28 | -8 | -20 | 5 |
|  | ER |  |  |  |  |  | 24 | 2 | -26 | 19 |
|  | JH | -12 | -2 | -26 | 7 |  | 20 | -4 | -22 | 19 |
|  | NL | -26 | -8 | -14 | 12 |  | 28 | -12 | -10 | 12 |
|  | NT | -22 | -6 | -25 | 14 |  | 24 | -8 | -23 | 9 |
|  | NW |  |  |  |  |  | 26 | -4 | -16 | 18 |
|  | ST | -22 | -6 | -16 | 15 |  | 24 | -2 | -18 | 10 |
|  | ZI |  |  |  |  |  | 22 | -2 | -16 | 9 |
|  | ZM |  |  |  |  |  | 24 | -10 | -10 | 6 |
|  |  |  |  |  |  |  |  |  |  |  |
| **LC** | BN |  |  |  |  |  | 4 | -36 | -34 | 6 |
|  | ER |  |  |  |  |  | 2 | -38 | -24 | 19 |
|  | JH |  |  |  |  |  | 2 | -26 | -46 | 14 |
|  | NL | -4 | -34 | -30 | 6 |  | 4 | -30 | -30 | 10 |
|  | NT |  |  |  |  |  | 5 | -16 | -12 | 16 |
|  | NW |  |  |  |  |  | 2 | -40 | -36 | 18 |
|  | ST |  |  |  |  |  | 4 | -32 | -30 | 6 |
|  | ZI | -6 | -34 | -16 | 10 |  | 0 | -36 | -44 | 6 |
|  | ZM |  |  |  |  |  | 4 | -18 | -18 | 17 |
|  | ZO | -12 | -9 | -27 | 5 |  | 9 | -9 | -27 | 14 |
|  |  |  |  |  |  |  |  |  |  |  |
| **PFC** | BN | -30 | 56 | 8 | 63 |  | 32 | 58 | -4 | 5 |
|  | EO | -18 | 64 | 12 | 109 |  | 10 | 64 | 8 | 63 |
|  | ER | -18 | 64 | -4 | 59 |  | 20 | 56 | 2 | 59 |
|  | JH |  |  |  |  |  | 4 | 42 | -8 | 44 |
|  | NL | -8 | 50 | 0 | 86 |  | 28 | 46 | 4 | 5 |
|  | NT | -12 | 47 | 0 | 72 |  | 14 | 58 | 14 | 4 |
|  | NW |  |  |  |  |  | 30 | 58 | 6 | 61 |
|  | ST | -8 | 36 | -10 | 109 |  | 10 | 52 | -12 | 6 |
|  | ZI | -24 | 44 | -2 | 32 |  | 10 | 58 | -2 | 38 |
|  | ZM |  |  |  |  |  | 30 | 48 | 10 | 14 |
|  | ZO | -24 | 63 | 9 | 24 |  | 21 | 63 | 6 | 14 |
