# Supplementary material for: Eyes Wide Shut: Amygdala Mediates Eyes-Closed Effect on Emotional Experience with Music
Source: PLoS One. 2009 Jul 15;4(7):e6230. doi: 10.1371/journal.pone.0006230 (PMC2705682; doi:10.1371/journal.pone.0006230)
Supplement: Table S2 — Regions of interest used in functional analysis. Size and location of regions of interest. The location is given as Talairach coordinates of the center of the region (X, Y, Z). (0.03 MB DOC) [file pone.0006230.s004.doc]

**Supplementary Table 2.** *Regions of interest used in functional analysis*.

| **Talairach coordinates (mm)** | | | | | | | | | |
| --- | --- | --- | --- | --- | --- | --- | --- | --- | --- |
|  | **Left hemisphere** | | | |  | **Right hemisphere** | | | |
| **Region of Interest** | **x** | **y** | **z** | **N of voxels** |  | **x** | **y** | **z** | **N of voxels** |
| Amygdala | -20+/-3 | -5+/1 | -12+/-2 | 273+/-70 |  | 20+/-2 | -4+/-1 | -13+/-2 | 355+/-95 |
| Hippocampus | -23+/-2 | -12+/-1 | -13+/-1 | 189+/-57 |  | 22+/-1 | -11+/-1 | -14+/-1 | 305+/-54 |
| BA 46/9 | -40+/-5 | 7+/-3 | 33+/-5 | 389+/-62 |  | 43+-5 | 9+/-6 | 24+/-3 | 408+/-82 |
| BA 47 | -37+/-6 | 30+/-5 | -3+/-4 | 966+/-90 |  | 35+/-5 | 33+/5 | -8+/-1 | 833+/-110 |
| BA 11 | -19+/-3 | 44+/-2 | -6+/-1 | 899+/-95 |  | 17+/-2 | 48+/-3 | -5+/-2 | 715+/-47 |
|  |  |  |  |  |  |  |  |  |  |
