# Supplementary material for: Eyes Wide Shut: Amygdala Mediates Eyes-Closed Effect on Emotional Experience with Music
Source: PLoS One. 2009 Jul 15;4(7):e6230. doi: 10.1371/journal.pone.0006230 (PMC2705682; doi:10.1371/journal.pone.0006230)
Supplement: Table S3 — Correlation coefficients. Correlation coefficients for each subject are presented for un-normalized time-courses. Co-activation of the amygdala with area BA 47 and co-activation of the amygdala with area BA 46/9 were computed. Activation in the BA 47 was obtained in contrast ‘close > open’ for the negative and neutral stimuli separately. Activation in the BA 46/9 was obtained in contrast ‘open > close’ for the negative and neutral stimuli separately. (0.05 MB DOC) [file pone.0006230.s005.doc]

**Supplementary Table 3.** *Correlation coefficients*

|  | **BA 47 and amygdala** | | | **BA 46/9 and amygdala** | | | **time points** |
| --- | --- | --- | --- | --- | --- | --- | --- |
|  | **negative** | **neutral** |  | **negative** | **neutral** |  |  |
| Sub1 | 0.2685 | 0.294 |  | 0.2911 | 0.02 |  | 279 |
| Sub2 | 0.3435 | 0.1026 |  | 0.1769 | 0.0888 |  | 279 |
| Sub3 | 0.175 | 0.4043 |  | 0.5718 | 0.2329 |  | 279 |
| Sub4 | 0.3467 | 0.3474 |  | -0.3907 | -0.0385 |  | 279 |
| Sub5 | 0.5179 | 0.6195 |  | 0.0961 | -0.1194 |  | 279 |
| Sub6 | 0.4291 | 0.3459 |  | -0.1023 | -0.2962 |  | 279 |
| Sub7 | 0.4964 | 0.4071 |  | 0.2458 | 0.3176 |  | 279 |
| Sub8 | 0.3717 | 0.1964 |  | 0.2136 | -0.0772 |  | 279 |
| Sub9 | 0.2356 | 0.0207 |  | 0.1044 | 0.1634 |  | 279 |
| Sub10 | 0.3655 | 0.3623 |  | 0.0696 | 0.0552 |  | 279 |
| Sub11 | 0.2599 | 0.2187 |  | 0.2153 | 0.1342 |  | 279 |
|  |  |  |  |  |  |  |  |
